# Walking the Hospital in Johannesburg

**Published:** 1919-11-29

**Authors:** 


### Walking the Hospital in Johannesburg.

AT a meeting last month of the Johannesburg Hospital Board the Rand University Council inquired if students of the Medical School, now being established at Johannesburg in connection with the University, would be admitted to the hospital for training. The Council explained that provision had been definitely made for the first two and a-half years' medical curriculum, and it was not anticipated that there would be any walking of the hospital before August 1921.

The Chairman informed the Board that there would be 563 free beds at the hospital almost immediately available for the teaching of students. The University Council's request was agreed to in principle by the Board.

